# Material Discrimination Based on K-edge Characteristics

**DOI:** 10.1155/2013/308520

**Published:** 2013-11-12

**Authors:** Peng He, Biao Wei, Peng Feng, Mianyi Chen, Deling Mi

**Affiliations:** ^1^The Key Lab of Optoelectronic Technology and Systems of the Education Ministry of China, Chongqing University, Chongqing 400044, China; ^2^The Key Lab of Biorheological Science and Technology of the Education Ministry of China, Chongqing University, Chongqing 400044, China

## Abstract

Spectral/multienergy CT employing the state-of-the-art energy-discriminative photon-counting detector can identify absorption features in the multiple ranges of photon energies and has the potential to distinguish different materials based on K-edge characteristics. K-edge characteristics involve the sudden attenuation increase in the attenuation profile of a relatively high atomic number material. Hence, spectral CT can utilize material K-edge characteristics (sudden attenuation increase) to capture images in available energy bins (levels/windows) to distinguish different material components. In this paper, we propose an imaging model based on K-edge characteristics for maximum material discrimination with spectral CT. The wider the energy bin width is, the lower the noise level is, but the poorer the reconstructed image contrast is. Here, we introduce the contrast-to-noise ratio (CNR) criterion to optimize the energy bin width after the K-edge jump for the maximum CNR. In the simulation, we analyze the reconstructed image quality in different energy bins and demonstrate that our proposed optimization approach can maximize CNR between target region and background region in reconstructed image.

## 1. Introduction

X-ray computed tomography (CT) has been widely applied in clinical and preclinical applications, since Hounsfield's Nobel Prize winning breakthrough. A typical conventional CT system employs a broad energy spectrum source and a digital integrating sensor whose output is proportional to the energy fluence integrated over the entire incidence spectrum. Physically, the X-ray spectrum contains much information; the conventional CT system collects photons over the whole X-ray spectrum to ignore spectral responses of materials. Hence, the conventional CT often does not have sufficiently high contrast resolution for biological soft tissues [1].

With the development of spectral detectors and novel contrast agents, CT image contrast resolution could be significantly improved. Recent advances in spectral/multienergy detector technology have allowed for spectral CT systems to identify absorption features in the multiple ranges of photon energies [2–6]. Spectral CT has a stronger capability to distinguish different materials because it can capture images in available energy bins [7–13]. Meanwhile, contrast agent has been widely applied in biomedical imaging to enhance tissue contrast [14–19]. Spectral CT imaging utilizes not only density characteristics of contrast agents but also K-edge characteristics of contrast agents to distinguish different materials. K-edge characteristics involve the sudden attenuation increase in the attenuation profile of some contrast agents, which could be captured by spectral CT in available energy bins. Hence, different materials can be easily distinguished according to their K-edges characteristics [20, 21], while their Hounsfield numbers may be very similar in conventional CT images. This opens a door for spectral CT to support functional, cellular, and molecular imaging studies.

For contrast agent imaging by spectral CT, threshold settings for available energy bins have a major impact on spectral image quality in terms of image contrast and noise level. Hence, it is important to partition the energy bin optimally based on K-edge characteristics. In this paper, we propose a contrast agent imaging model to optimize energy bin for maximum material discrimination with spectral CT. Based on this model, we investigate how to select one energy bin for optimal contrast agent imaging to distinguish different materials, introducing a contrast-to-noise ratio (CNR) where the signal difference is defined between contrast enhancement region (CER) and background region values. 

This paper is organized as follows.  Section 2 introduces our proposed contrast agent imaging model.  Section 3 describes our simulation experiment.  Section 4 demonstrates our experimental results.  Section 5 discusses relevant issues and concludes the paper.

## 2. Materials and Methods

An earlier paper [22] has introduced the theoretical formalism of K-edge imaging model to determine two energy bins on both sides of the K-edge and analyzed the effect of K-edge energy bins on the resultant image quality. Here, we only briefly reproduce the optimization scheme and analyze how to optimize one energy bin after the K-edge jump to distinguishing contrast enhancement region and background region.

For current spectral CT system, its spectral detector (e.g., Medipix-3 [4–6]) is a photon-counting system with selectable thresholds, which depends on a threshold equalization mask to adjust each pixel to record different energy photons. We assume that the energy distribution function of an X-ray source is *I*_0_(*E*); in a given energy threshold *T*, we have the photon number received by the spectral detector:

(1) $$\begin{array}{c} I_{T}\left(E\right)=\int_{T}^{\infty }I_{0}\left(E\right)\eta \left(E\right)dE, \end{array}$$

where *η*(*E*) is the detector efficiency.

For a given energy bin defined by two energy thresholds 0 < *T*_1_ < *T*_2_, the received photon number can be expressed as

(2) $$\begin{array}{c} I_{\left(T_{1},T_{2}\right)}\left(E\right)=\int_{T_{1}}^{T_{2}}I_{0}\left(E\right)\eta \left(E\right)dE. \end{array}$$

This paper focuses on how to set the thresholds in the energy bin imaging to distinguish contrast enhancement region and background region based on K-edge characteristics. First, we need to study the linear attenuation characteristics of background materials and contrast agents. Let *a*_*B*_(*E*) be the linear attenuation coefficient function of a background material at an energy *E*; we have

(3) $$\begin{array}{c} a_{B}\left(E\right)=\sigma_{B}\left(E\right)\rho_{B}; \end{array}$$

we assume that *a*_*C*_(*E*) is the linear attenuation coefficient function of a contrast agent at an energy *E*, and *a*_*C*_(*E*) can be expressed as

(4) $$\begin{array}{c} a_{C}\left(E\right)=\omega \sigma_{C}\left(E\right)\rho_{C}+\left(1-\omega \right)\sigma_{B}\left(E\right)\rho_{B}, \end{array}$$

where *σ*_*B*_(*E*) and *σ*_*C*_(*E*) are the mass attenuation coefficients of background material and contrast agent, *ρ*_*B*_ and *ρ*_*C*_ are the densities of background material and contrast agent, respectively, and *ω* is the concentration of the contrast agent.

**Figure 1:**
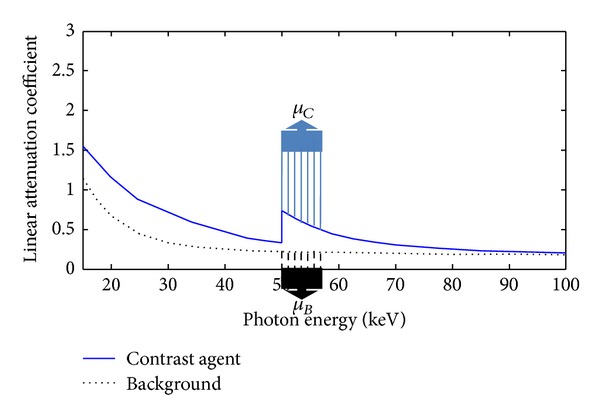
Attenuation profiles of a typical contrast agent and a soft tissue (background material).

For a given contrast agent concentration *ω*, we plot two linear attenuation profiles of a typical contrast agent and background material (i.e., tissue) according to their mass attenuation coefficients and densities, which are shown as in Figure 1. In Figure 1, the attenuation coefficients of the contrast agent have a sudden increment at an energy *K*, which reflects the K-edge characteristics. Theoretically, if we perform an energy bin imaging at the point *K* with spectral CT, there is a maximum material discrimination for background region and contrast agent region in reconstructed image. However, the narrower the energy bin width is, the higher the noise level is, and there are few photons to carry the information. Hence, we select one energy bin of finite width to study contrast agent imaging in this paper. Let ${\overset{-}{\mu }}_{B}$ be the average attenuation coefficient of the background material within the energy bin after the K-edge jump; we have

(5) $$\begin{array}{c} {\overset{-}{\mu }}_{B}=\frac{1}{w}\int_{K}^{K+w}a_{B}\left(E\right)dE=\frac{1}{w}\int_{K}^{K+w}\sigma_{B}\left(E\right)\rho_{B}dE, \end{array}$$

where *w* is the energy bin width, and let ${\overset{-}{\mu }}_{C}$ be the average attenuation coefficient of the contrast agent within the energy bin after the K-edge jump; we have

(6) μ−C=1w∫KK+waC(E)dE=1w∫KK+w(ωσC(E)ρC+(1−ω)σB(E)ρB)dE.

For a given energy bin after the K-edge jump, from (2) we have the received photon number

(7) $$\begin{array}{c} I_{w}\left(E\right)=\int_{K}^{K+w}I_{0}\left(E\right)\eta \left(E\right)dE. \end{array}$$

The reconstructed images can be evaluated as contrast-to-noise ratio (CNR), and the CNR can be defined as

(8) $$\begin{array}{c} \text{CNR}=\frac{{\bar{\mu }}_{C}-{\bar{\mu }}_{B}}{\sqrt{\phi_{C}^{2}+\phi_{B}^{2}}}, \end{array}$$

where *ϕ*_*C*_^2^ and *ϕ*_*B*_^2^ are corresponding variances of the contrast agent region and background region in reconstructed image. 

The difference between the mean value of contrast agent region and background region relies on the energy bin width *w* and contrast agent concentration *ω*. For a given reconstructed object, the variances of reconstructed image rely on the photon number *I*_*w*_(*E*) determined by the energy bin width *w*. Hence, we can search for the optimal *w* value to maximize the CNR for maximum material discrimination. In the following, we will make numerical simulation to test the proposed imaging model, including phantom design and image reconstruction protocols.

## 3. Numerical Simulation

**Figure 2:**
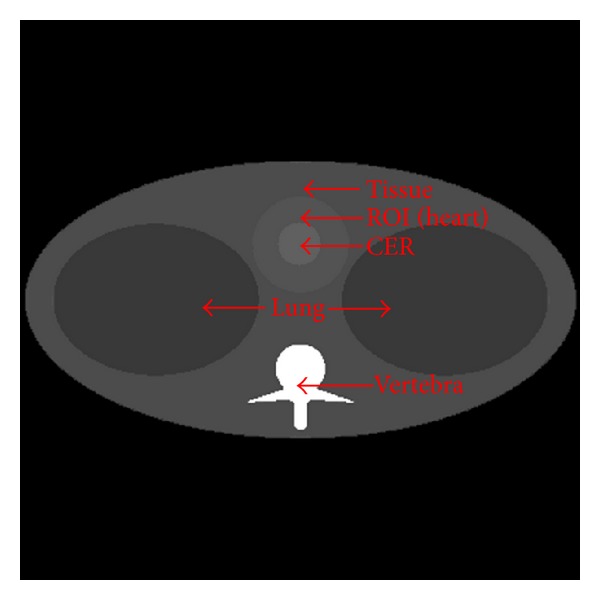
Thorax phantom.

In the simulation, a thorax phantom (Figure 2) was designed to be more preclinically relevant, which is defined on http://www.imp.uni-erlangen.de/forbild/. The phantom contains a heart region, a tissue region, a lung region, a vertebra region, and a contrast enhancement region (CER). The phantom was made 25 cm × 25 cm in size and discretized into a 500 × 500 matrix. We used Gadolinium solution whose K-edge is 50 keV, as a testing contrast agent in the CER inside the heart region, and the whole heart region is considered as the region of interest (ROI). 

To investigate the proposed imaging theory, we study how to search for the optimal energy bin to maximize the CNR for maximum material discrimination. First, we study the characteristics of the thorax phantom materials. For tomographic imaging, the linear attenuation coefficient *μ* represents the gray value of reconstructed image. Here, we can obtain the mass attenuation coefficient *μ*/*ρ* according to the X-ray attenuation databases reported by the National Institute of Standards and Technology (NIST). To calculate the linear attenuation coefficients of the phantom materials, the densities *ρ* of these materials were selected in reference to the biomedical literature [23–26] and summarized in Table 1. In our simulation, we used blood attenuation characteristics to substitute heart attenuation characteristics.

**Figure 3:**
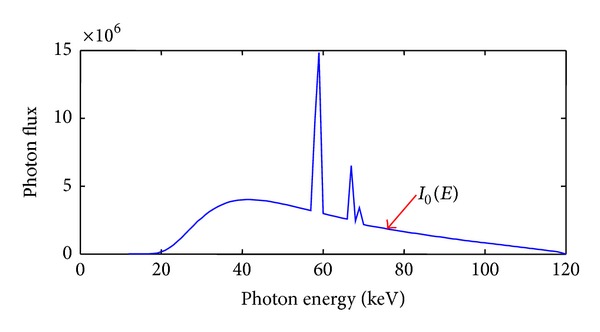
Source photon emission spectra.

Then, we used a free-of-charge software program (SpekCalc) [27] to calculate X-ray spectra from tungsten anode tubes. The X-ray tube voltage is assumed as 120 kVp with a 2.5 mm Al filter, and its emission spectra are shown in Figure 3. In our study, it was assumed that the detector efficiency *η*(*E*) was 90%, the spectral CT system was viewed as in a typical parallel-beam geometry, and the scanning range was from 0° to 180° with a 1° angular increment in the given energy bins. According to the Beer-Lambert law, we can capture the thorax phantom projection data.

To perform the energy bin imaging with spectral CT, we used a typical analytical reconstruction protocol: filtered backprojection (FBP), and a reconstructed image using FBP formula can be expressed as

(9) $$\begin{array}{c} f\left(x,y\right)=\int_{0}^{\pi }d\theta \int_{+\infty }^{-\infty }g\left(t^{\prime }\right)h\left(t-t^{\prime }\right)dt^{\prime }, \end{array}$$

where *g* = ∫_*L*_ *μ*(*w*, *l*)*dl* is the integral of the linear attenuation coefficient distribution along an X-ray path. 

Then, we can calculate the expected image *f*(*x*, *y*)

(10) E(f(x,y))=E(∫0πdθ∫+∞−∞g(t′)h(t−t′)dt′)=∫0πdθ∫+∞−∞g(t′)h(t−t′)dt′,

and the variance of reconstructed image *f*(*x*, *y*) [22, 28]

(11) Var⁡(f(x,y))=Var⁡(∫0πdθ∫+∞−∞g(t′)h(t−t′)dt′)=∫0πdθ∫+∞−∞1Iwe−g(t′)h2(t−t′)dt′.

From (10) and (11), CNR of ROI in reconstructed image can be written as follows:

(12) CNR(w) =(∫0πdθ∫+∞−∞gC(w,t′)h(t−t′)dt′       −∫0πdθ∫+∞−∞gB(w,t′)h(t−t′)dt′)  ×(∫0πdθ∫+∞−∞1Iwe−gC(w,t′)h2(t−t′)dt′        +∫0πdθ∫+∞−∞1Iwe−gB(w,t′)h2(t−t′)dt′)−1/2,

where *g*_*C*_(*w*, *t*) is the reconstructed sinogram of the contrast agent region and *g*_*B*_(*w*, *t*) is the reconstructed sinogram of the background material region. From (12), we can find that the CNR will depend on the energy bin width *w*. In Section 4, we will calculate the best energy bin width *w* value after the K-edge jump to maximize the CNR of ROI.

## 4. Results

**Figure 4:**
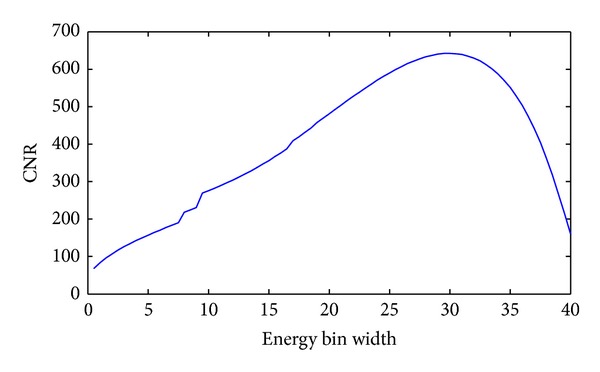
Relationship between the energy bin width (*w*) and CNR. The curve for Gadolinium solution (0.5%) in the thorax phantom.

**Figure 5:**
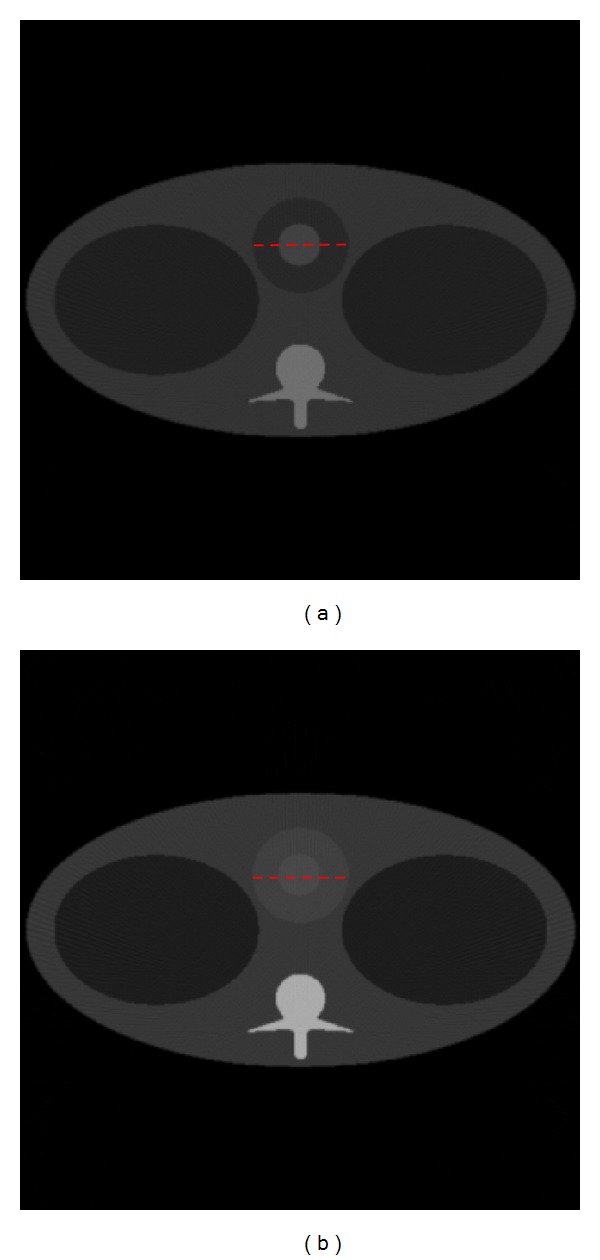
Two model imaging results. (a) is the reconstructed thorax phantom image in the optimal energy bin, and (b) is the reconstructed thorax phantom image in a broad energy spectrum. The display window for the two images is [0, 1].

**Figure 6:**
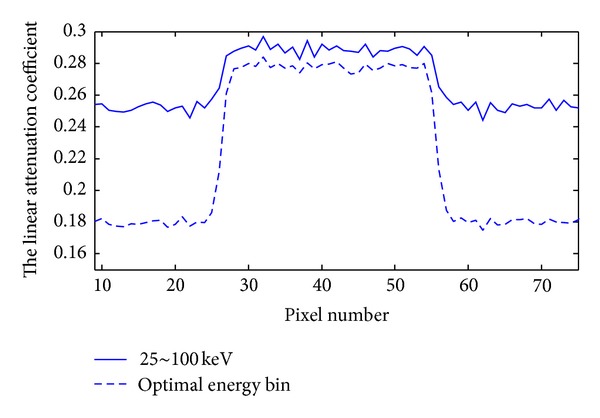
Profiles corresponding to the broken lines in Figure 5.

We used our proposed approach to analyze the thorax phantom and determined the best energy bin for imaging based on K-edge characteristics and plotted the relationships between *w* and CNR, as shown in Figure 4. Then, we used the optimal *w* value (29 keV) to perform energy bin imaging for the thorax phantom, and the reconstructed image is shown in Figure 5(a). Meanwhile, we chose a broad energy spectrum (25~100 keV) to reconstruct the thorax phantom which can be considered as the conventional CT imaging, and the reconstructed result is shown in Figure 5(b). Compared to the broad energy spectrum imaging result, it is easier to distinguish Gadolinium solution region and heart region in optimal energy bin imaging result. Finally, we calculated the CNR of ROI in Figures 5(a) and 5(b), which is summarized in Table 2. From Table 2, we can see that CNR of ROI in Figure 5(a) is smaller than that in Figure 5(b). To compare the reconstructed results, we plotted the profiles along the broken lines in Figure 5, as shown in Figure 6.

**Figure 7:**
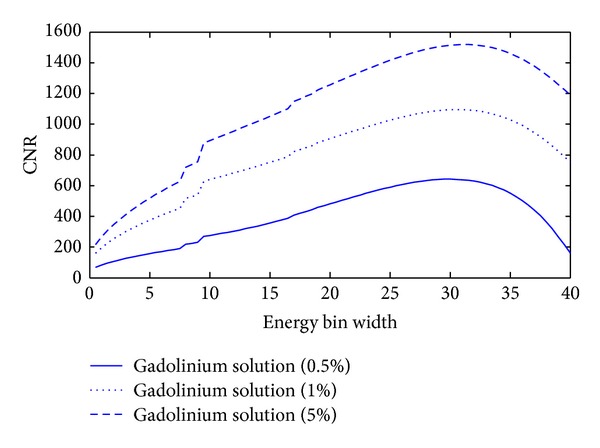
Relationships between the energy bin width (*w*) and CNR. The curves for Gadolinium solutions (0.5%, 1%, and 5%) in the thorax phantom.

The CNR of ROI also relies on the contrast agent concentration *ω*, and then we analyze the relationships between CNR and the concentration *ω* of contrast agent. We chose different concentrations of Gadolinium solution (0.5%, 1%, and 5%) as the testing contrast agents and plotted the relationships between *w* and CNR with different concentrations, as shown in Figure 7. Finally, we calculated the optimal *w* and maximum CNR for different concentration contrast agents, and the results are summarized in Table 3. From Table 3, we can see that the higher the concentration of Gadolinium solution is, the wider the optimal energy bin width is, and the bigger the CNR of ROI is.

## 5. Discussions and Conclusion

This paper is a follow-up study for an earlier paper [22]. Although some relevant theories are similar, this study focuses on how to distinguish contrast agents and background materials (i.e., tissue) in biomedical imaging with spectral CT, which can be readily generalized to deal with more general settings and able to determine the best energy bin for maximum material discrimination.

There are several issues worth further discussion in the simulation. First, we apply a mimetic X-ray emission spectrum in our study; the X-ray emission spectra with 1 keV energy bins are obtained by the free-of-charge software program (SpekCalc). To analyze relationships between energy bin *w* and CNR of ROI, we calculate the photon number *I*_*w*_(*E*) with the given energy bin width *w* value of an integer. As a result, it is inevitable to introduce some errors, compromising the estimation of the optimal energy bin width *w*. Second, the curves of CNR in Figure 7 are not so smooth in some energy bins, which reflects the real CNR characteristics in the given X-ray emission spectra. The unsmooth reason is that the X-ray emission spectra have some drastic jumps in some energies. If the imaging energy bin contains these drastic jumps, the photon number will drastically increase, and the variance of ROI in the reconstructed image will drastically decrease, so the curves of CNR in Figure 7 have some jumps in corresponding energy bins. Additionally, the proposed approach depends on specific phantom configurations, and the optimal energy bin width is application-specific. Nevertheless, our optimization theory is rigorous and can be applied once the application context or the class of images is known. In a follow-up study, we will study the biomedical samples with the spectral CT based on our proposed imaging theory.

In conclusion, contrast agent imaging with spectral CT has a great potential for clinical applications including, but not limited to, tissue characterization and contrast studies. We proposed a contrast agent imaging model to optimize energy bin for maximum material discrimination; it established guidelines for optimization of energy thresholds and could be readily generalized for biomedical imaging.

## Figures and Tables

**Table 1:** Biologically relevant densities of phantom materials.

Materials	Blood	Bone	Lung	Tissue
Density (g/cm^3^)	1.05	1.9	0.26	1.0

**Table 2:** Summary of maximum CNR for different imaging models.

Imaging models	CNR
Optimal energy bin (50~79 keV) imaging	642.1
Broad energy spectrum (25~100 keV) imaging	438.0

**Table 3:** Summary of the optimal width and maximum CNR for different concentration contrast agents.

Contrast agents	Optimal *w* (keV)	Maximum CNR
Gadolinium solution (0.5%)	29	642.1
Gadolinium solution (1%)	30	1084.9
Gadolinium solution (5%)	32	1519.0
